# Multimodal processing in simultaneous interpreting with text: Evidence from ear-eye-voice span and performance

**DOI:** 10.1371/journal.pone.0326527

**Published:** 2025-07-03

**Authors:** Shanshan Yang, Defeng Li, Victoria Lai Cheng Lei

**Affiliations:** 1 Department of English, Central China Normal University, Wuhan, Hubei Province, China; 2 Department of English, University of Macau, Macao SAR, China; University of Missouri Columbia, UNITED STATES OF AMERICA

## Abstract

Simultaneous interpreting (SI) with text, a hybrid modality combining auditory and visual inputs, presents greater cognitive complexity than traditional SI. This study investigates multimodal processing in Chinese-English SI with text by examining how source speech rate and professional experience modulate interpreters’ Ear-Eye-Voice Span (EIVS)—a temporal measure reflecting the cognitive coordination among auditory input, visual processing, and verbal output—and interpreting performance. Using eye-tracking technology, we analyzed EIVS patterns in 15 professional interpreters and 30 interpreting trainees performing three SI with text tasks at slow, intermediate and fast speech rates. EIVS measures, including Ear-Eye Span (EIS), Eye-Voice Span (IVS), and Ear-Voice Span (EVS), were analyzed to assess temporal coordination of listening, reading and interpreting processes. Results indicate that faster speech rates significantly reduced EIVS across all measures, suggesting accelerated information processing and strategic cognitive adaptation. A significant interaction effect between speech rate and professional experience was observed. Professionals maintained more stable and efficient EIVS patterns, particularly under accelerated speech rates, reflecting an advantage in cross-modal attention allocation and cognitive resource management. In contrast, trainees exhibited greater reliance on visual input, and struggled more with multimodal demands, manifested in longer EIVS values and greater individual variation. Both groups exhibited an ear-lead-eye coordination pattern during the fast speech rate task, though professionals achieved more efficient auditory-visual synchronization. Despite a decline in interpreting performance with increasing speech rates, professionals consistently outperformed trainees. These findings underscore the critical role of experience in enhancing multimodal coordination, and highlight the importance of dedicated skill-specific practice in enhancing auditory-visual coordination and optimizing interpreting performance under cognitively demanding conditions.

## Introduction

Simultaneous interpreting (SI) is described as “one of the most complex language tasks imaginable because many processes take place at the same time” [1, p.454], requiring interpreters to manage multiple tasks: listening to the source speech, producing the target output, and monitoring their own delivery. The increasing prevalence of visual materials in international conferences—from PowerPoint slides and speakers’ notes, to real-time automatic speech recognition (ASR) or machine translation (MT) speech transcripts—reflects the growing trend toward multimodal interpreting [2–4]. As one UN interpreter remarked, “much of our work here at UNHQ involves sight translation of texts read at speed by delegates” [2, p. 64]. With fast speech rates and tight schedules becoming the norm, interpreters must efficiently coordinate auditory, visual, and verbal processing under significant time pressure [5]. Consequently, the ability to synchronize these inputs has become a critical skill for professional interpreters navigating increasingly complex multimodal environments.

Time lag measures offer valuable insights into the cognitive dynamics of multimodal input management [6–10]. Traditional temporal metrics such as Ear-Voice Span (EVS) capture the time delay between hearing the source and delivering the target. In multimodal contexts, Ear-Eye Span (EIS) captures the timing of auditory and visual processing coordination, while Eye-Voice Span (IVS, also a notable measure in sight translation and consecutive interpreting) captures the delay between an interpreter’s visual fixation and target output production. Together, these measures—collectively referred to as Ear-Eye-Voice Span (EIVS)—offer a comprehensive framework for studying multimodal cognitive coordination in interpreting [11–13]. Each measure maps onto specific cognitive processes. EIS reflects cross-modal attention and resource prioritization strategies; IVS indicates the efficiency of visual-to-verbal transformation, including lexical access and syntactic planning; and EVS represents the entire processing chain from input reception to output delivery. Collectively, EIVS enables analysis of how interpreters manage multimodal demands [14,15], providing a nuanced understanding of how interpreters manage the demands of multimodal input during interpreting.

Despite growing interest in multimodal processing, the complexities of SI involving visual inputs remain relatively underexplored [12,16]. Specifically, little is known about how interpreters coordinate visual and auditory inputs, or how such patterns influence performance. Theoretical models such as Multiple Resource theory [17] and working memory constructs [18] provide valuable insights into cognitive resource distribution across modalities. Meanwhile, Gile’s Effort Model [8] and Seeber’s Cognitive Load Model [19] explain how increased task complexity elevates cognitive processing load [20,21] and impacts temporal coordination. Building on these frameworks, EIVS provides a robust tool to examine how interpreters dynamically allocate cognitive resources during multimodal processing, particularly under high-demand conditions where efficient management is critical for successful performance.

This study investigates EIVS in Chinese-English SI with text, focusing on how variations in source speech rate—a well-documented challenge in conference interpreting [8,11]—modulate cognitive coordination and performance. Existing research suggests that professional interpreters demonstrate greater flexibility and efficiency in managing multimodal inputs, allowing for more effective temporal synchronization under pressure [11]. By comparing professionals and trainees, this study explores how experience shapes EIVS patterns and interpreting performance, offering insights into the developmental trajectory of cognitive processing in interpreting and its relationship to professional expertise [6,11,22].

Using eye-tracking technology to analyze gaze behavior and ear-eye-voice coordination patterns, we systematically investigate how EIVS interacts with multimodal inputs and affects interpreting performance across varying source speech rates. The present study contributes to the growing body of research on multimodal interpreting, offering nuanced insights into the cognitive mechanisms underlying Chinese-English SI with text. It demonstrates how EIVS measures capture distinct aspects of temporal coordination shaped by speech rate and interpreter experience. As the interpreting landscape becomes increasingly multimodal, with visual input and fast-paced delivery becoming the norm, these findings may inform training strategies aimed at balancing auditory and visual inputs, helping to equip interpreters with the cognitive flexibility needed to perform effectively in high-pressure, multimodal settings.

## Multimodal processing in simultaneous interpreting

Information is rarely transmitted via a single modality in real-world communication. As Seeber notes [23], multimodal processing, involving the integration of information across sensory modalities, constitutes a defining characteristic of SI, where interpreters must process multiple inputs simultaneously. Two representative theoretical accounts, Gile’s Effort Model [8] and Seeber’s Cognitive Load Model [23] have significantly informed our understanding of multimodal processing in interpreting. Gile conceptualizes interpreting as a competition for limited cognitive resources across listening, memory, and production efforts. The presence of visual inputs introduces an additional reading effort, potentially leading to cognitive saturation [24,25]. Thus, interpreters must continually recalibrate their cross-modal resource distribution in response to task demands.

Seeber [23] builds on Wickens' Multiple Resource theory and conceptualizes multimodal interpreting complexity through a conflict matrix that accounts for overlapping sensory demands. He quantifies cognitive load by an interference matrix, highlighting the substantial resource conflict in SI with text scenarios, necessitating skilled coordination to prevent cognitive strain. Together, these frameworks underpin current inquiry into how EIS, IVS, and EVS map onto distinct cognitive processes in multimodal SI.

In SI with text, interpreters process visual input from the source text and auditory input from the speaker that may be congruent or occasionally divergent [24]. This dual-channel input demands that interpreters cautiously allocate scarce processing capacity to comprehend and reformulate information in real time—a challenge exacerbated when modalities compete for shared linguistic resources [20,25]. For example, reading while listening taxes cognitive resources and can lead to what Gile terms “cognitive saturation” [8, p.192].

Empirical studies present mixed evidence on the role of visual input in SI. Congruent multimodal inputs have been shown to support comprehension and improve interpreting performance [11,26–32]. For instance, Korpal and Stachowiak-Szymczak [11] reported that professional interpreters rendered numerical data more accurately when supported by PowerPoint slides, particularly at higher speech rates. This aligns with Gile’s [8] argument that reading effort complements listening effort under congruent conditions, reducing memory load and acoustic strain. Novice interpreters also benefit from congruent visual input, as Yang et al. [32] demonstrated, where visual support helped mitigate challenges caused by rapid delivery. Similarly, Lambert [31] and Desmet et al. [28] reported improved number accuracy and fewer omissions. These findings validate Seeber’s conflict matrix, where visual input functions as a cognitive aid or “friend” [33, p.130] rather than a competitor.

However, visual input does not universally confer advantages. While text can facilitate comprehension, it may also challenge ear-eye-voice coordination [34]. Baxter noted that visual input may distract interpreters, as they must analyze the “consistency” [35, p.10] between audio-visual messages, potentially increasing EVS. Chen [27] observed that professional interpreters sometimes forgo textual support in high-complexity tasks, as reconciling conflicting information may strain cognitive resources. This strategic avoidance reflects an adaptive effort rebalancing—reducing reading load to optimize listening-production balance. Chmiel et al. reported that incongruent cognates introduced additional cognitive strain and linguistic interference, with eye-tracking data showing prolonged visual fixation on incongruent items to resolve modality conflicts [5, p.48] despite decreased accuracy. Seeber [23] reported that visual information significantly increased task conflict by occupying both “visual-verbal” and “visual-spatial” resources, exacerbating conflicts with auditory modalities. Such paradoxes underscore the temporal cost of modality conflict, the finite nature of cognitive resources, and the trade-offs interpreters face in prioritizing competing modalities [8,20].

Expertise plays a critical role in managing multimodal processing demands. Research consistently shows that professional experience significantly influences interpreters’ attention allocation patterns [11,13]. Seubert [36] highlighted that professional interpreters prioritize critical information across modalities, strategically disregarding redundant visual cues to prevent cognitive strain. Chmiel et al. [37] observed that professional interpreters tended to process auditory inputs first in SI with text tasks, though they also devoted considerable visual attention to texts in cases of incongruent auditory-visual inputs, while student interpreters demonstrated more rigid reading patterns, with longer fixation durations and lower interpreting accuracy under similar conditions. Korpal and Stachowiak-Szymczak [11] found that professional interpreters utilized textual aids to resolve ambiguities in complex numerical data and maintained high accuracy even at rapid speech rates. While such strategies demand considerable visual processing effort, professionals consistently outperformed student interpreters, reflecting greater processing efficiency. Notably, professional interpreters exhibited shorter fixations than students, indicating more efficient visual information extraction. Similarly, Stachowiak-Szymczak and Korpal [26] reported that student interpreters exhibited more and longer fixations when faced with sophisticated visual input conditions. This aligns with Gile’s [8, p.182] observation that novices often feel compelled to rely on text because, “its content remains available in ‘solid’ print whereas words disappear rapidly”. Collectively, these findings highlight the nuanced expertise-related differences in multimodal processing efficiency, underscoring expertise’s impact on cognitive resource management and interpreting performance.

Despite these insights, significant gaps remain in understanding multimodal processing in SI. Seeber et al. [12] highlighted limited knowledge of visual processing in SI with text, while Yuan & Wang [16] noted sporadic research on interpreters’ attentional preferences in multimodal contexts. Although auditory input is traditionally prioritized in SI [15,24], reflecting practical communicative context demands, recent evidence reveals professionals’ covert reliance on visual cues under stress [5]. Similarly, Baxter [35] found that interpreters’ attention shifted toward visual slides based on auditory cues. Seeber et al. [12] also observed a notable ear-lead-eye processing pattern in SI with text, where interpreters relied on visual input to support interpreting production rather than processing the speaker’s auditory input. These tensions underscore the need for further exploration of how interpreters coordinate multimodal inputs under varying task conditions, particularly in high-pressure scenarios that characterize professional interpreting environments.

### Time lag in interpreting studies

Time lag measures, including EIS, IVS and EVS, serve as sensitive and quantifiable indicators of cognitive processing traits and coordination patterns in multimodal interpreting [6,8–10,38,39]. These measures are particularly valuable for their ability to quantify variations in task conditions and interpreter expertise, offering a framework for studying cognitive processes in interpreting [40]. In SI with text, EIVS offers valuable insights into how auditory and visual inputs are coordinated [5,34].

Grounded in Gile’s Effort Model [8] and Seeber’s Cognitive Load Model [19], each EIVS component can be mapped onto specific cognitive processes involved in multimodal interpreting. EIS reflects attention distribution between auditory and visual channels during comprehension—“the interpreters” attention toward the ear or the eye [13, p.8]. IVS indicates the efficiency of converting visual input into verbal output, serving as an index of visual–verbal transformation [37,41]. EVS embodies the delay from auditory comprehension to speech production, reflecting an interpreter’s ability to balance concurrent processing demands between input and output [10]. Together, these measures reveal both processing efficiency and strategic adaptations of interpreters to task demands during multimodal interpreting tasks. While EVS is well studied in traditional SI, IVS and EIS, particularly in multimodal settings, remain underexplored.

Table 1 summarizes representative studies on time lag in interpreting. Longer EVS typically reflects greater processing pressure and task demands, and reduced performance [9,42]. Chang [42] observed that EVS exceeding 5 seconds correlated with increased disfluencies, errors and omissions in Chinese-English SI. Similarly, Collard and Defrancq [6] reported that longer EVS is associated with increased production effort in SI, indicated by longer filled pauses and more false starts. This supports Gile’s concept of “cognitive saturation,” [8, p.192] where excessive listening or reading efforts deplete resources from production effort.

**Table 1:** A brief review of representative literature on time lag in interpreting.

Scholars	Working modes	Variables	Sample	Findings
[30] 2001	SI with/without text	EVS	12 PR	longer EVS for SI with text EVS = 2.5–10.2 seconds
[9] 2002	SI	EVS	8 PR	Average EVS = 3 seconds; Longer EVS (>4 seconds) associated with low accuracy output
[42] 2009	C-E SI	EVS	5 students	EVS(>5 seconds) associated with most omissions
[7] 2015	SI	EVS	PR-corpus	Shorter EVS for cognates
[41] 2018	sight translation with 3-minute preparation	EVS	24 students	Average EVS = 3.02 seconds before metaphorical expressions for students
[6] 2019	SI	EVS	78 females 79 males	Longer EVS associated with delivery disfluency markers and declined interpreting quality EVS female M = 3.01 seconds; male M = 3.05 seconds
[32] 2020	SI SI with text	EVS	54 NV	SI: EVS M = 2.821 seconds SI with text: EVS M = 3.32 seconds
[44] 2021	sight translation	IVS	30 students	Longer eye-voice span associated with longer total fixation duration E-C M = 2.16650 seconds; F-C-E M = 1.90912 seconds
[43] 2020	sight translation	IVS	14 students	Mean overall Max. IVS= 3.47 seconds; L1 shorter IVS than L2
[34] 2022	Sight translation	IVS	24 PR	Mean IVS exceeding 8 seconds
[13] 2022	E-C SI with text	EIS, IVS, EVS	9 PR	IVS M = 2.187 seconds; EVS M = 6.002 seconds; EIS M = −4.400 seconds

In English to Korean SI, sentences with EVS above 4 seconds demonstrated lower quality than sentences with EVS below 2 seconds [9]. In sight translation, longer EVS has also been linked to challenges processing complex items, like metaphorical expressions, which require more cognitive resources [41,45]. Conversely, shorter EVS is generally associated with enhanced proficiency and efficient information processing [9], particularly under faster source delivery rates. Chmiel et al. [22] noted that shorter EVS promotes fluency and temporal coordination and reduces disfluency markers in high-demand scenarios. De Groot [39] also recommends that interpreters keep EVS short to facilitate faster processing and optimize performance. These findings support the negative correlation between EVS duration and interpreting performance in traditional SI contexts.

However, studies of SI with visual inputs reveal a more nuanced role of EVS. With visual information available, longer EVS can provide interpreters with additional time to align auditory and visual inputs, thereby improving accuracy and coherence [13]. This dual role of EVS—balancing fluency against multimodal alignment—echoes Seeber’s conflict matrix [23], where added visual input increases both interference risk and compensatory support. For instance, visual input in SI with text may extend EVS as interpreters balance reading effort with listening and production efforts, yet this prolonged EVS does not necessarily impair performance and may even improve interpreting quality, as demonstrated by Lamberger-Felber [30]. These findings challenge the view that shorter EVS invariably benefits performance and emphasize task-specific strategic adaptations. Furthermore, professional interpreters exhibit greater flexibility in adapting EVS to task demands. De Groot [39] and Timarová et al. [45] suggest that interpreters can extend EVS in certain scenarios to improve alignment in multimodal tasks or shorten it to maintain fluency in fast-paced interpreting. These patterns demonstrate professionals’ strategic effort rebalancing to avoid cognitive saturation [8].

Although less studied than EVS, IVS and EIS provide critical insights into visual-auditory coordination [10,34,46,47]. Zou et al. [13] identified an ear-lead-eye processing pattern in SI with text, where interpreters often begin producing target speech before fully processing visual input, as evidenced by an average EIS of −4400 milliseconds. This negative time lag, though seeming relatively large, underscores the cognitive demands of SI with text in contrast to the visual dominance in reading-while-listening tasks [12]. Zou et al. [13] further suggest that EIS reflects interpreters’ general preference for eye or ear in interpreting, revealing their ability to dynamically prioritize multimodal information. Chmiel et al. [34] further found that shorter IVS often correlates with more efficient visual-verbal processing, while longer IVS may indicate increased cognitive effort or deliberate strategic adjustments. These findings provide valuable insights into how interpreters coordinate visual and auditory inputs during multimodal interpreting tasks, revealing the complex interplay between different cognitive processes involved in multimodal integration.

Despite advancements, key gaps remain in understanding time lag dynamics, particularly for IVS and EIS in multimodal interpreting scenarios. Existing studies offer limited insight into how dynamic task variables, such as speech rate, modulate temporal coordination and interpreting performance. The present study aims to address these gaps by investigating how variations in source speech rate modulate EIVS patterns in Chinese-English SI with text and examining the association between these temporal dynamics and interpreting performance. By systematically exploring these relationships, we aim to deepen our understanding of the cognitive mechanisms underlying multimodal interpreting and identify strategies for improving interpreter training and performance. The following research questions are proposed:

1. How does source speech rate modulate the temporal dynamics of Chinese-English interpreters in SI with text, as observed in EIS, IVS, and EVS?
2. What is the association between temporal dynamics and interpreting performance?

## Methods

This study employs a 2 × 3 mixed factorial design, where each participant completed SI with text tasks under three speech rate conditions, while a between-group comparison was made between professional interpreters and interpreting trainees. The study was approved by the ethical committee of the School of Foreign Languages at Central China Normal University and conducted from March 1, 2021 to December 30, 2023. All participants (see Table 2 for Participants profiles) voluntarily signed written informed consent before participating in the study.

**Table 2:** Participants profiles.

Group	Number	Age	Language background	Interpreting experience
**Trainees**	30 (22F, 8M)	22-29, M = 24.9 (1.13)	L1-Chinese, L2-English	M = 0.1 year (1.3)
**Professionals**	15 (11F, 4M)	26-42, M = 31.2 (3.69)	L1-Chinese, L2-English	M = 8.06 years (3.86)

### Participants

The analysis included 30 interpreting trainees (TR) and 15 professional interpreters (PR). All participants had normal or corrected-to-normal vision, with Chinese Putonghua as their first language (L1) and English as their second language (L2). The trainees (aged 22–29, M = 24.9, SD = 1.13, 22 females and 8 males) were enrolled in the Master of Translation and Interpreting Program at the University, with one semester of SI training. Only some trainees had occasional liaison or on-campus/remote SI practices. The 15 professional interpreters (aged 26–42, M = 31.2, SD = 3.69, 11 females and 4 males) all received systematic interpreting training at Master level or higher, with an average of eight years of practice (threshold set at four years) working as freelance (11) or in-house interpreters (4) in China. It should be noted that five of the freelance professionals also teach interpreting at universities.

### Experiment design

The experiment (see Table 3) consisted of three Chinese-to-English SI with text tasks at three speech rates. Each speech rate was paired with a fixed Chinese source text, while the order of the three tasks was randomized across participants to control for order effects. All interpreters completed a 2-minute Chinese shadowing task for warm-up, which was not included in the final analysis. The source texts for interpreting tasks were displayed on a 23-inch eye-tracker monitor (resolution 1024 × 768, Imitated Song typeface, Font size 20, double spaced), occupying one full screen. Eye-tracking data were collected via the Tobii TX300 eye-tracker at a sampling frequency of 300 Hz. Participants sat approximately 60 cm from the eye-tracker to ensure optimal gaze data quality, listening to the speaker through a headset. No preparation was allowed, ensuring that performance reflected real-time cognitive processing. After the interpreting tasks, a retrospective interview and questionnaire survey were conducted.

**Table 3:** Experiment design.

TASK ID	TASK description	Participants	TASK SEQUENCE
TASK 1	C-E SI with text, slow speech rate	30 TR, 15 PR	Randomized
TASK 2	C-E SI with text, intermediate speech rate	30 TR, 15 PR	Randomized
TASK 3	C-E SI with text, fast speech rate	30 TR, 15 PR	Randomized

### Stimuli control

Three non-domain-specific texts were adapted from speeches by former Chinese state leaders delivered to university students. To ensure research validity and minimize the introduction of confounding variables [48], the three stimuli texts were carefully calibrated to ensure comparable complexity across tasks [49,50] at lexical, syntactic and delivery levels (see Table 4). Additionally, we invited 10 bilingual experts (aged 20–35, M = 30.7, SD = 4.57), all native Chinese speakers with MA or higher degrees in translation and interpreting disciplines to rate the texts on a 7-point Likert rating scale across seven dimensions, reading difficulty, interpreting difficulty, lexical complexity, syntactic complexity, information density, logic complexity, and language conciseness. These experts were familiar with the working mode of interpreting and capable of identifying key challenges related to interpreting complexity. Their ratings indicated no significant differences among the texts.

**Table 4:** Source text complexity control description.

Category	Item	Task 1	Task 2	Task 3
Lexical parameters	Word count (character)	200	200	200
	Unrepeated words ratio	0.74	0.72	0.71
	Lexical density	0.78	0.76	0.78
	Difficult words	31	33	31
	Non-literalness	1	1	1
Syntactic parameters	Number of sentences	15	14	15
	Characters per sentence	13.33	14.28	13.33
	Complex sentences	11	10	10
Delivery parameters	Duration	85.10 seconds	55.30 seconds	42 seconds
	Speech rate (syllables per minute)	141	218	285
Bilingual expert assessment results	7 dimensions: Reading difficulty, interpreting difficulty, lexical complexity, syntactic complexity, information density, logic complexity, and language conciseness.	3.75/7	3.9/7	3.80/7

Given the Chinese language specificity and the lack of a widely recognized standard for ideal speech rate [51], referring to the proposed “optimal” Chinese speech rate of 150–180 syllables per minute by Li [52], we instructed the speaker to maintain a natural delivery flow while targeting three distinct speech rates: approximately 140 syllables, 210 syllables and 300 syllables per minute, respectively. The final recordings used for the tasks were slow (141 syllables/minute, Task 1), intermediate (218 syllables/minute, Task 2), and fast (285 syllables/minute, Task 3). The speeches were recorded by a young male speaker with professional training in Chinese public speaking. Retrospective feedback confirmed the speaker’s standard pronunciation and steady pace. Participants also reported no specific textual challenges across the tasks, with speech rate serving as the primary differentiating factor.

### Data collection and processing

The data collected included eye-tracking data, interpreting recordings, and retrospective questionnaire results. Given the focus of this research on the temporal dynamics of cognitive processing, particularly the EIVS patterns, the retrospective questionnaire data were not included in the current analysis.

### EIVS processing

**Fig 1:**
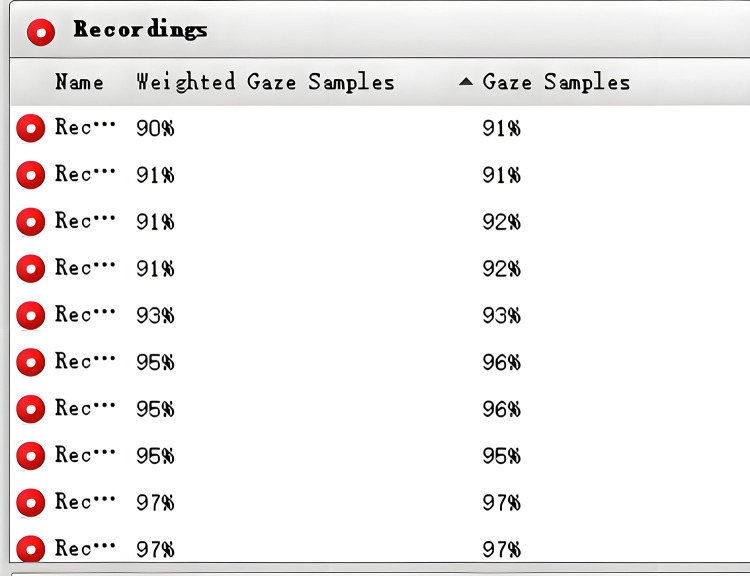
Weighted gaze samples in Tobii Studio.

Eye-tracking data and interpreting recordings were synchronized from the same starting point to enable precise temporal alignment. Eye-tracking data quality was rigorously scrutinized through established procedures [53,54], considering factors such as Weighted gaze samples, Gaze duration, and Gaze Time on Screen. First, eye-tracking data were considered valid if at least 80% of Weighted gaze samples (see Fig 1) were retained, with Gaze duration accounting for at least 50% of the total task duration. Both Weighted gaze samples and Gaze duration were provided by Tobii Studio, the eye-tracker’s data processing software. High Gaze time on screen suggests that the participant devoted considerable time looking at the screen, or that the eye tracker effectively recorded the participant’s eye movements. These thresholds were determined based on prior studies demonstrating that such levels of data validity and gaze engagement are sufficient for reliable analyses of gaze behavior in translation and interpreting tasks [53,54].

Additionally, the manual cross-check was implemented to ensure that the majority of fixations fell on the screen. This step allowed for accurate gaze-to-word mapping—namely, to map specific fixations on certain words in the source text [55] by gaze plot analysis. The I-VT fixation filter in Tobii Studio sets the default minimum fixation duration threshold at 60 milliseconds. We further followed the suggestion to apply “the minimum fixation duration of 100 milliseconds” [56, p.65], to distinguish meaningful fixations from rapid saccades or incidental glances in high-demand tasks like SI, where rapid gaze-switching is common. While traditional fixation metrics such as fixation count and fixation duration are commonly reported in eye-tracking studies, these metrics are not relevant to the current research focus. Originally, 35 trainees and 17 professionals participated in the experiment. After evaluating the eye-tracking data, 30 trainees and 15 professionals were included in the analysis.

**Fig 2:**
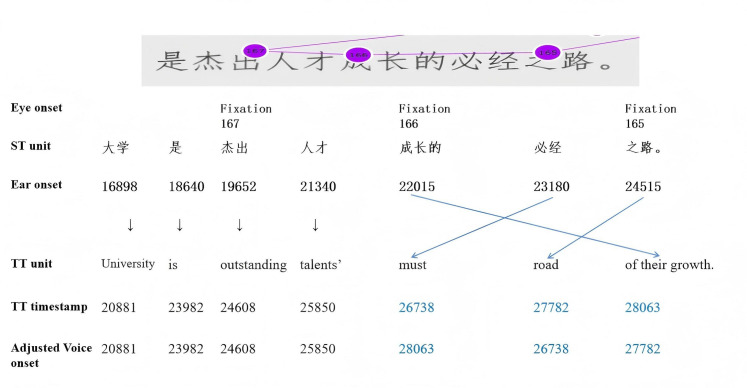
Visualization of EIVS calculation.

To integrate eye-tracking and timestamped interpreting recordings, this study followed Carl et al. [57], using source text and target text tokens (words) as the basic analytical segment (see Fig 2), such as nouns (e.g., 大学-University) or adjectives (e.g., 杰出-outstanding). EIVS was calculated as the three time lag measures between the interpreter’s first fixation on a source text token (Eye onset), the corresponding timestamps of speech onset by the speaker (Ear onset), and the onset of the corresponding target text token (Voice onset). The IBM Watson Speech-to-Text platform (https://www.ibm.com/products/speech-to-text) was used to generate timestamped ASR transcriptions of the interpreting recordings. These transcriptions and corresponding timestamps (in milliseconds) were later manually verified using ELAN software.

A challenge in analyzing EIVS is linguistic restructuring that occurs during the interpreting process, which can alter grammatical sequences and structures in the interpreting output. For instance, in Fig 2, the source text token “大学” corresponds to the target text token “university”. The sequence “成长的必经之路” (which can be idiomatically translated as “the essential path for their growth”) in the source speech was literally interpreted as “the must road (必经之路) of their growth (成长的)”, showing a reversed token order compared to the source text. Accordingly, we made minor adjustments to the target text token sequence, which were strictly limited to the influenced sequence alignment (see the time stamps in blue in Fig 2) and did not alter the content or meaning of the interpreted source speech. Another emerging issue is the presence of missing values in some datasets, such as source speech segments without corresponding reading times (like “人才” and “必经”) or interpreting output. As the number of incomplete values was minimal upon comprehensive evaluation, segments with missing values were excluded from analysis. The mean values of EIS, EVS, and IVS were subsequently used as input data for the linear mixed-effects regression analysis.

### Interpreting performance indicators

The analysis of interpreting performance included both experts’ holistic assessment and quantitative analysis of interpreting output at content and delivery levels (see Table 5). Following Kahane [58], two expert interpreters and trainers rated the interpreting performance on a 10-point Likert scale. The average values were used for analysis. Their inter-rater reliability demonstrated a high level of agreement (Krippendorff’s α = 0.93 for trainees and 0.95 for professionals).

**Table 5:** Details of Interpreting performance analysis.

Interpreting performance	Codes	Description
Holistic assessment	**SCORE**	10-point Likert scale, assessed by the two expert raters
Content level	**LD**	calculating the percentage of content words against the number of tokens
	**SSN**	mutual agreement on qualified sentences by the two expert raters
	**ASL**	ASL = total word count of SSN/number of SSN
Delivery level	**ISR**	syllables/min, pause included
	**IFP**	the frequency of filler utterance
	**IPD**	The total duration of silent pause

Research has shown that interpreters often produce shorter words and sentences to manage the multitasking demands of simultaneous interpreting, especially under cognitive pressure [46]. Consequently, sentence length and lexical density offer critical insights into interpreters’ cognitive management strategies and delivery. Therefore, in the content-level interpreting output analysis, the Target Text Lexical Density (LD), Number of Successfully Rendered Sentences (SSN), and Average Sentence Length (ASL) were considered. Delivery level analysis referred to relevant established practices [59,60] and focused on the Interpreter’s Speech Rate (ISR), Interpreter’s Pause Duration (IPD, threshold at 0.3 seconds) and Interpreter’s Filler Frequency (IFP), all of which are widely recognized as indicators of fluency and temporal coordination. Together, these measures reflect the interpreters’ capacity to produce accurate and fluent output, offering an indirect yet robust assessment of interpreting quality.

### Statistical analysis

The research results were analyzed using the lme4 package [61] in R Studio version 4.3.0 to perform a linear mixed-effects regression analysis on the relationship between source speech rate variance and the EIVS pattern of interpreters. This statistical method was chosen for its merits of including both fixed and random effects in the model [62]. Three LMER models were built, with Speech Rate (SR) and Interpreter Group (GROUP) as fixed effects, and participants entered as random effects. The dependent variables for the three models were the three temporal measures: the EIS, IVS and EVS. Model 1 examined the effect of speech rate variance on EIS, Model 2 on IVS, and Model 3 on EVS. These analyses tested for main effects and interactions of the fixed effects (SR and GROUP) on each of the individual measures of EIVS. The results of the regression models are presented in Table 8 in the Results section.

## Results

This section provides an overview of the descriptive statistics of EIVS, the results of the interpreting performance indicators analysis, and the effect of source speech rate on EIS, IVS and EVS.

### Descriptive statistics: EIVS and interpreting performance

**Fig 3:**
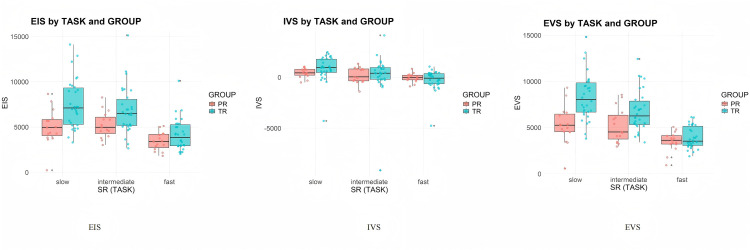
Professionals and trainees' EIVS values in the three tasks (3a-EIS, 3b-IVS, and 3c-EVS).

Table 6 presents descriptive statistics for EIS, EVS and IVS across the three tasks for both groups (see Fig 3), reported in average values (M) and standard deviations (SD). All three measures exhibited a clear declining trend as source speech rate increased. Professionals consistently demonstrated shorter and more stable EIVS values compared to trainees. For EIS and EVS, trainees had larger temporal distances and greater variability across tasks. Notably, both groups displayed negative IVS values in Task 3, with professionals showing less pronounced deviations.

**Table 6:** Descriptive Statistics of EIVS.

ITEM	TASK 1	TASK 2	TASK 3
EIVS TYPE	M (SD) (milliseconds)	M (SD) (milliseconds)	M (SD) (milliseconds)
EIS [TR]	7545.94 (2712.91)	6837.38 (2610.66)	4173.44 (1773.74)
EIS [PR]	5003.32 (2002.97)	5244.96 (1472.76)	3408.83 (1007.71)
IVS [TR]	840.44 (1257.62)	203.87 (2024.20)	−285.65 (1037.60)
IVS [PR]	440.24 (464.58)	200.87 (763.01)	−49.58 (43.60)
EVS [TR]	8333.41 (2551.20)	6795.92 (2230.70)	3908.24 (1191.35)
EVS [PR]	5307.87 (2101.44)	5241.37 (1871.63)	3500.05 (1095.08)

**Fig 4:**
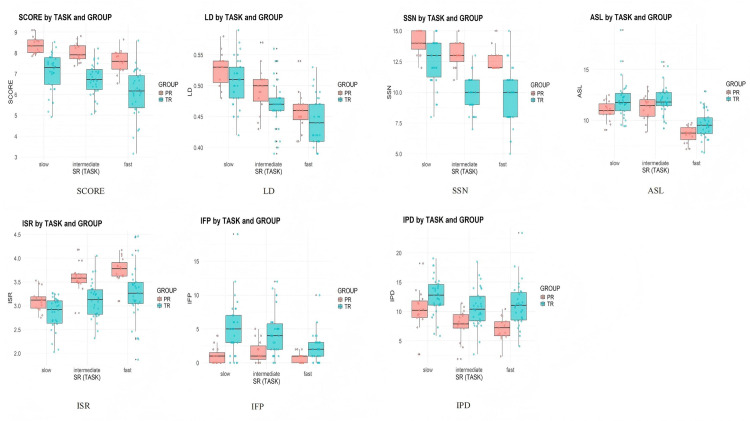
Professionals and trainees' performance indicators in the three tasks (4a-SCORE, 4b-LD, 4c-SSN, 4d-ASL, 4e-ISR, 4F-IFP, 4g-IPD).

Table 7 provides descriptive statistics for interpreting performance indicators (see Fig 4). As the speech rate increased from Task 1 to Task 3, both groups showed a decline in performance, lexical density, successfully rendered sentences, and average sentence length, indicating that higher speech rate negatively affected content richness and structural complexity. In contrast, the delivery fluency indicators such as interpreter speech rate improved with faster speech rates, while disfluency markers, including pause duration and filler frequency, decreased. Professionals consistently outperformed trainees across all performance indicators, showing better fluency, fewer disfluency markers, and greater stability. Trainees exhibited greater variability and had more difficulty maintaining their performance under higher speech rates.

**Table 7:** Descriptive Statistics of Interpreting Performance Indicators.

ITEM		TASK 1	TASK 2	TASK 3
GROUP/PERFORMANCE		M (SD)	M (SD)	M (SD)
TR	SCORE (out of 10)	7.08 (0.91)	6.72 (0.76)	6.08 (1.17)
	LD (percentage)	0.51 (0.04)	0.47 (0.04)	0.44 (0.04)
	SSN (out of 15)	12.5 (1.87)	9.76 (1.43)	9.73 (2.27)
	ASL (word count)	12.04 (1.93)	12.01 (1.45)	9.56 (1.43)
	ISR (syllables/second)	2.84 (0.35)	3.11 (0.39)	3.27 (0.58)
	IFP (frequency)	5.03 (4.14)	4.33 (3.28)	2.2 (2.09)
	IPD (second)	12.69 (3.24)	10.65 (3.36)	11.13 (3.81)
PR	SCORE (out of 10)	8.39 (0.41)	8.08 (0.39)	7.61 (0.52)
	LD (percentage)	0.53 (0.03)	0.49 (0.03)	0.46 (0.03)
	SSN (out of 15)	14.13 (0.99)	12.98 (1.01)	12.53 (0.88)
	ASL (word count)	10.97 (0.96)	11.29 (1.19)	8.62 (0.8)
	ISR (syllables/second)	3.11 (0.24)	3.58 (0.28)	3.76 (0.26)
	IFP (frequency)	1 (1.25)	1.73 (1.57)	0.73 (0.77)
	IPD (second)	10.39 (3.39)	7.78 (2.53)	7.09 (1.94)

### Effect of speech rate and professional experience on EIVS

Table 8 presents the summary results of the linear mixed-effects regression models fitted to EIS, IVS and EVS, respectively.

**Table 8:** Summary results of linear-mixed effects regression models fitted to EIVS (P-values are marked with the symbol* for significant effects).

EIVS type	Predictors	Estimate	CI	p
EIS	GROUP [PR]	6744.79	4720.58-8769.00	<0.001*
	GROUP [TR]	10822.76	9391.43-12254.10	<0.001*
	SR	−9.97	−18.19- −1.74	0.018*
	GROUP× SR	−11.11	−21.19- −1.03	0.031*
	Marginal R^2^/ Conditional R^2^	0.293/0.521		
IVS	GROUP [PR]	870.70	−38.10–1779.51	0.060
	GROUP [TR]	1801.25	1158.63–2443.88	<0.001*
	SR	−3.06	−6.13–0.01	0.051
	GROUP× SR	−3.98	−7.74 – −0.22	0.038*
	Marginal R^2^/ Conditional R^2^	0.088/ 0.742		
EVS	GROUP [PR]	7168.84	5204.82–9132.96	<0.001*
	GROUP [TR]	12430.23	11041.46–13819.00	<0.001*
	SR	−11.30	− 19.53 – −3.07	0.008*
	GROUP× SR	−16.36	−26.44 – −6.28	0.002*
	Marginal R^2^/ Conditional R^2^	0.430/ 0.526		

### EIS results

**Fig 5:**
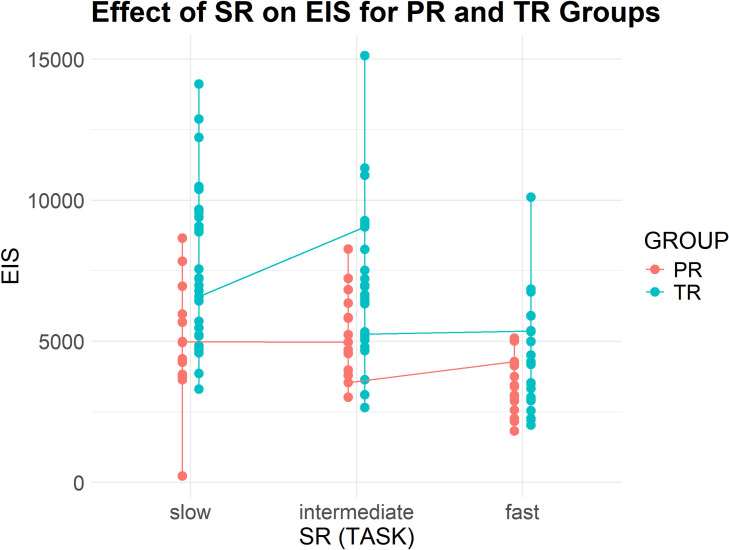
Effect of Group and SR on EIS.

EIS (see Fig 5) demonstrated a marked reduction for both groups as speech rate increased from Task 1 to Task 3. For trainees, the mean EIS dropped from 7545.94 milliseconds (SD = 2712.91) in Task 1 to 4173.44 milliseconds (SD = 1773.74) in Task 3. Professionals exhibited consistently shorter EIS values (ranging from 3400 to 5000 milliseconds), declining from 5003.32 milliseconds (SD = 2002.97) in Task 1 to 3408.83 milliseconds (SD = 1007.71) in Task 3.

Regression results show that both fixed effects (Group and SR) had a significant impact on EIS. First, SR had a significant negative effect (Estimate = −9.97, CI [−18.19, −1.74], p = 0.018), indicating more efficient coordination between listening and reading as speech rate increased. GROUP was also a significant predictor for EIS, with professionals (Estimate = 6744.79, CI [4720.58, 8769.00], p < 0.001) exhibiting significantly shorter EIS values than trainees (Estimate = 10822.76, CI [9391.43, 12254.10], p < 0.001). Additionally, a significant interaction effect between GROUP and SR (Estimate = −11.11, CI [−21.19, −1.03], p = 0.031) highlighted that speech rate influenced EIS more strongly for trainees than for professionals. Overall, the model explained 52.1% of the variance accounting for both fixed and random effects in the EIS data. These results demonstrate that as speech rate increases, both professionals and trainees adjust their cross-modal attention allocation strategies, reducing the time lag between auditory input reception and visual information localization. However, professionals demonstrate more stable coordination patterns across different speech rates, suggesting more efficient and adaptive cross-modal attention allocation strategies.

### IVS results

**Fig 6:**
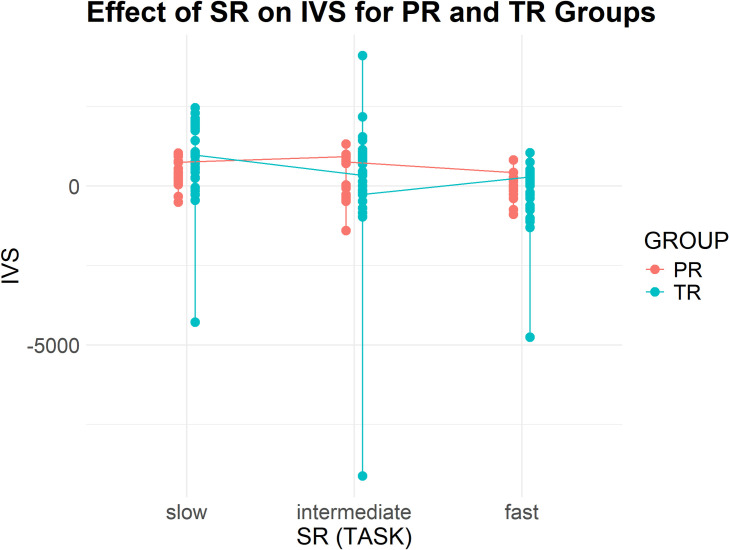
Effect of Group and SR on IVS.

IVS (see Fig 6) also showed a decline for both groups. For trainees, the mean IVS dropped from 840.44 milliseconds (SD = 1257.62) in Task 1 to −285.65 milliseconds (SD = 1037.60) in Task 3. For professionals, IVS dropped from 440.24 milliseconds (SD = 464.58) in Task 1 to −49.58 milliseconds (SD = 43.60) in Task 3, indicating better synchronization of visual and verbal processing. Although the negative IVS in Task 3 suggests an ear-lead-eye pattern for both groups, where interpreters begin producing target speech before fully processing the corresponding visual information, professionals demonstrated better synchronization of visual and auditory processing.

The fixed effect of GROUP was not significant on IVS for professionals (Estimate = 870.70, CI [−38.10 to 1779.51], p = 0.060). However, the GROUP effect was significant for trainees (Estimate = 1801.25, CI [1158.63, 2443.88], p < 0.001). SR had a non-significant negative effect on IVS (Estimate = −3.06, CI: [−6.13, 0.01], p = 0.051), suggesting that changes in speech rate do not significantly alter IVS across groups. A significant interaction effect between GROUP and SR was observed (Estimate = −3.98, CI [−7.74, −0.22], p = 0.038), indicating that speech rate had a greater impact on trainees’ IVS than on professionals. Overall, the model explained 74.2% of the variance, accounting for both fixed and random effects in the IVS results.

The IVS results demonstrated efficient eye-voice coordination for both groups, with mean values consistently falling within one second across all three conditions. This suggests efficient transformation from visual input processing to verbal output production. The emergence of negative IVS under high speech rates reflects strategic adaptation to task demands, as interpreters prioritize maintaining temporal alignment with the speaker over complete visual processing. This adaptation appears more controlled among professionals, who demonstrate less extreme negative IVS values, suggesting more precise anticipatory processing rather than compromised comprehension.

### EVS results

**Fig 7:**
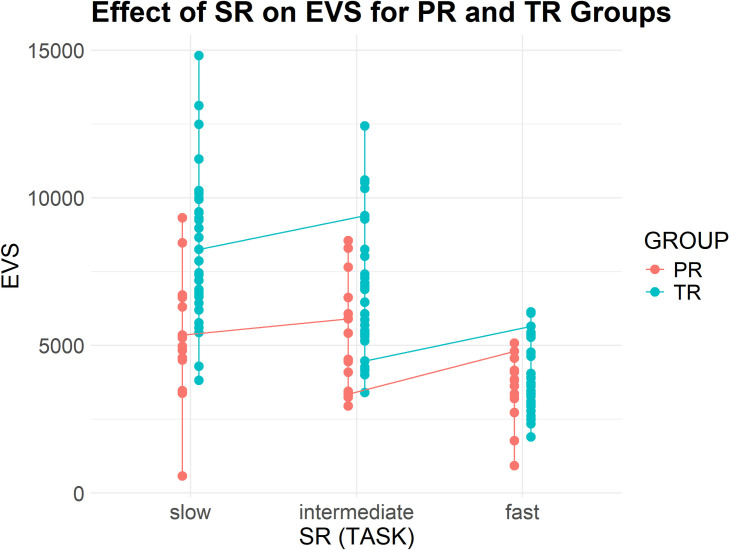
Effect of Group and SR on EVS.

EVS results (see Fig 7) decreased significantly as speech rate increased, indicating faster auditory-verbal coordination for both groups. For trainees, the mean EVS dropped from 8333.41 milliseconds (SD = 2551.20) in Task 1 to 3908.24 milliseconds (SD = 1191.35) in Task 3. Professionals consistently maintained shorter EVS values, declining from 5307.87 milliseconds (SD = 2101.44) in Task 1 to 3500.05 milliseconds (SD = 1095.08) in Task 3. At the same time, both groups showed similar EIS-EVS patterns across the three conditions, with the mean time gap being less than one second. Professionals maintained EVS within the normal range in the slow speech rate task (M = 5307.87, SD = 2101.44), as suggested by previous studies, while trainees exhibited notably longer EVS (M = 8333.41, SD = 2551.20), suggesting greater difficulty in balancing listening and production efforts.

SR had a significant negative effect on EVS (Estimate = −11.30, CI [−19.53, −3.07], p = 0.008), while GROUP showed significant differences between professionals (Estimate = 7168.84, CI [5204.82, 9132.96], p < 0.001) and trainees (Estimate = 12430.23, CI [11041.46, 13819.00], p < 0.001). A significant interaction effect between GROUP and SR (Estimate = −16.36, CI [−26.44, −6.28], p = 0.002) suggested that SR had a more pronounced impact on reducing EVS for trainees than for professionals. Overall, the model explained 52.6% of the variance accounting for both fixed and random effects in the EVS results. These findings indicate that both groups adapt their processing pace to match speech rate, but professionals demonstrate more controlled adaptation, reflecting their ability to efficiently manage cognitive resources under varying task demands and maintain more optimal balance.

## Discussion

This study examined how source speech rate modulates interpreters’ ear-eye-voice coordination patterns and performance in Chinese-English SI with text. Results confirm that accelerated speech rates significantly affect both cognitive processes and interpreting output. A notable interaction between speech rate and professional experience was observed, with professionals demonstrating shorter and more stable EIVS spans across all conditions. Both groups exhibited reduced EIS, IVS and EVS as source speech rates increased, along with declining content-based assessments despite improved fluency. Professionals consistently outperformed trainees across all tasks, underscoring that experience is critical for developing the cognitive capacity for efficient multimodal processing.

### Effect of source speech rate on EIVS

Research Question One addressed source speech rate variation effects on interpreters’ ear-eye-voice coordination patterns. The decline in EIVS values underscores strategic adaptations interpreters make to manage increasing cognitive demands. Rather than simply indicating accelerated processing pace, these temporal adaptations reflect sophisticated cognitive strategies that interpreters employ to maintain performance under pressure. Professionals displayed less variability and shorter temporal spans, particularly under higher speech rates, indicating greater cognitive flexibility and more efficient resource allocation. Trainees exhibited longer temporal spans and more variable patterns, highlighting their greater reliance on visual input and challenges in managing multimodal demands.

These results align with previous research identifying fast speech rates as a major challenge in interpreting [8,11,32], where increased cognitive demands necessitate strategic adaptations in ear-eye-voice coordination [9,21,27,32,45]. Our findings demonstrate how interpreters dynamically reallocate cognitive resources to balance competing efforts [8,19]. Notably, the emergence of an “ear-lead-eye” pattern at accelerated speech rates signals a strategic shift toward prioritizing auditory input over visual cues, enabling interpreters to cope with rapid delivery more effectively. This shift reflects an active attempt to redistribute cognitive resources to manage competing demands of listening, reading, and production, optimizing what Gile terms “processing capacity management” [8, p.185]. By accelerating output and minimizing lag, interpreters reduce short-term memory load and optimize performance under time pressure [13,19]. The transition from integrated multimodal processing at slow rates to selective attention at faster rates illustrates a fundamental change in strategy in response to increased task complexity.

Fast speech rates require interpreters to strategically prioritize information processing. According to Seeber [19], temporal synchronization is vital for interpreters to navigate the cognitive challenges of multimodal processing. In this study, at slower speech rates, interpreters, particularly trainees, relied more heavily on visual input in Task 1. However, as speech rate increased, interpreters gradually shifted to auditory-dominant processing, reflected in shortened EIS, IVS, and EVS. This transition illustrates interpreters’ dynamic adjustments to meet task demands [5,8], reinforcing the role of adaptive multimodal strategies as a critical component of interpreting performance [8,19,35]. Professionals, in particular, show their expertise-specific cognitive advantages with more efficient cross-modal scheduling (shorter EIS), faster visual-to-verbal transformation (stable IVS) and optimized throughput (controlled EVS reduction). This finding extends our understanding of how interpreters allocate attention resources under varying temporal constraints.

EIS results revealed that interpreters relied on visual input for comprehension at slower speech rates, with a gradual shift to auditory-dominant processing as source speech rate increased. This pattern aligns with Seeber’s Cognitive Load Model [19], which posits that in multimodal tasks, increased task pressure compels cognitive resources to shift toward the dominant channel. Average EIS values were positive in all three conditions, indicating interpreters’ sequential processing of multimodal inputs and active searching for visual information across various speech rates. Professionals consistently maintained shorter EIS values across all tasks, particularly at fast speech rates (3408.83 milliseconds for professionals and 4173.44 milliseconds for trainees), highlighting their efficient processing of multimodal inputs and ability to minimize delays. Trainees, however, exhibited longer EIS values, particularly at slower speech rates, reflecting greater dependence on visual input, consistent with Baxter’s [35] observation that novice interpreters allocate significant cognitive resources to visual information at the expense of auditory input. The decline in EIS values among trainees at faster speech rates suggests a gradual adaptation to auditory-dominant strategies, albeit less efficiently than professionals.

The average IVS values in this study (all less than one second) differ slightly from previous studies, such as about 2–3 seconds in sight translation [41,43], and approximately 2.187 seconds in SI with text [13]. These shorter IVS values suggest more immediate synchronization of visual and auditory inputs in SI with text, influenced by real-time interpreting demands. Under fast speech rate conditions, IVS results demonstrated an ear-lead-eye pattern [8,13,32], with negative IVS values indicating that interpreters began producing target speech before fully processing corresponding visual input segments. This strategy reflects interpreters’ reliance on auditory cues to maintain delivery under time constraints, as they adjust their temporal coordination to align with the speaker’s pace [12,22,32]. Professionals exhibited smaller negative IVS values and greater stability across tasks, suggesting more efficient cognitive processing and the ability to dynamically prioritize auditory input while compensating for incomplete visual processing [12,13,35]. In contrast, trainees displayed higher variability in IVS values, with greater reliance on visual input, reflecting developmental challenges in managing multimodal demands [12]. This dependence may hinder their ability to adapt to temporal constraints imposed by faster speech rates.

For EVS, both groups showed significant declines as speech rates increased. Shorter EVS durations reflect faster auditory-to-output coordination, reducing processing pressure and supporting fluent delivery [8]. The average EVS for professional interpreters in this study is consistent with previous research [9,10]. However, trainees exhibited longer EVS durations, particularly in Task 1 (T1: 8333.41 milliseconds, with one extreme case of 14.8 seconds for one participant). This suggests a greater reliance on visual information at the expense of real-time processing efficiency [8]. Eye-tracking and interpreting process playback reveal that this particular participant fell several sentences behind the speaker and relied heavily on reading as the main source of information. While in Task 3 the fast speech rate did prompt trainees to speed up, the overall processing pattern remained consistent, with a focus on reading over listening. This contradicts the professional convention of prioritizing auditory input [15,24,51], typical in SI with multimodal input where interpreters may require more time to align visual information with auditory input [32]. Baxter [35] explains that under non-challenging conditions, visual modality input can divert cognitive attention from auditory processing. Another explanation by Gile [8] is that students often attempt to sight translate visually presented textual information due to its immediate availability, which may lead to cognitive saturation. Therefore, Gile [8] suggests that maintaining SI with text performance requires practicing processing capacity management and maintaining an appropriate balance between listening and reading inputs, a skill that develops with professional experience. These findings align with previous research indicating that professional interpreters can better adapt to challenging task conditions due to their refined cognitive strategies and extensive experience [6,13,19].

The results highlight a clear professional advantage in managing EIVS dynamics. Professionals’ shorter and more stable spans reflect efficient allocation of cognitive resources, prioritization of auditory input, and the ability to maintain delivery under challenging speech rate conditions. This supports Setton and Dawrant’s [15] observation of experienced interpreters’ advantage in balancing multimodal inputs to optimize performance. While professionals also utilize visual input in SI with text [40], they appear more proficient in keeping EIVS short, as suggested by De Groot [39]. Their ability to dynamically adjust processing pace underscores greater cognitive flexibility and expertise in multimodal integration [8,11,19]. In contrast, trainees’ greater variability in EIVS measures reflects struggles in adapting to increased cognitive demands of fast speech rates. Their reliance on visual input, particularly at slower speech rates, highlights the challenges they face in achieving efficient multimodal integration [25].

### Association between EIVS and interpreting performance

Research Question Two explored the association between EIVS and interpreting performance. This study demonstrates that as speech rates increase, both groups experienced declines in holistic assessment scores, content completeness and accuracy, with fewer and shorter successfully rendered sentences, while delivery fluency indicators showed notable improvement. These findings reveal interpreters’ strategic trade-off between content accuracy and delivery fluency under heightened cognitive demands [7]. Notably, professionals consistently outperform trainees, demonstrating greater capacity for adaptation and resource optimization under fast speech conditions.

The strong link between interpreters’ ear-eye-voice coordination patterns and their interpreting performance underscores the cognitive strategies interpreters employ to manage task demands. At slow speech rates, both groups demonstrated the longest EVS durations alongside the best interpreting performance and content quality. These findings align with previous research [30,32], that identified a positive association between EVS duration and interpreting performance in SI with visual assistance. While longer EVS is typically associated with greater processing effort and potential disruptions in auditory-visual coordination, it also provides interpreters with additional time to integrate multimodal inputs effectively [13,34,43]. Therefore, the extended EVS in Task 1 likely reflects interpreters’ ability to effectively integrate multimodal inputs when cognitive demands are lower [20].

As source speech rate increased, EIVS durations shortened, accompanied by a decline in overall performance and content quality, as evidenced by decreases in lexical density, the number of successfully interpreted sentences, and average sentence length. According to Dual Task Interference Theory, which posits that tasks competing for overlapping cognitive resources can lead to performance trade-offs, interpreters seem to produce less sophisticated words and shorter sentences to cope with task demands [46]. Notably, professionals consistently outperformed trainees, particularly under fast speech conditions. In Task 3, professionals successfully rendered an average of 12.53 out of 15 sentences, compared to 9.73 for trainees. This substantial difference underscores the challenges trainees face in maintaining accuracy and information completeness under heightened demands, as excessive reliance on visual input may disrupt their temporal coordination, consistent with Baxter’s [35] findings. As discussed above, conversely, professionals demonstrated more stable and complete performance, reflecting their ability to dynamically adjust processing strategies to minimize dual-task interference and optimize capacity management [19].

The observed decline in EIVS durations and interpreting performance, particularly under fast speech conditions, underscores interpreters’ efforts to minimize temporal delays and reduce cognitive processing pressure [22,39]. This adaptation aligns with Gile’s Effort Model [8], which posits that increased task complexity compels interpreters to prioritize critical tasks, often at the expense of secondary processing demands. While the information completeness and accuracy were hampered as speech rate increased, reductions in pause durations and filler occurrences, combined with increased delivery rate, indicated improved delivery fluency [6,60]. In Task 3, a significant reduction in fillers occurred for both groups, and overall pause duration also significantly decreased. Even with congruent text available, our study provides evidence that shifts toward auditory-dominant processing with shorter EIVS under fast speech rates contributed to enhanced processing efficiency and delivery fluency [9,22]. These findings also align with Yang et al. [32], who highlighted the trade-offs between fluency and content completeness and accuracy under high speech rate conditions.

EIS trends offer additional insights into the interplay between visual processing and content completeness and accuracy. At slower speech rates, interpreters demonstrated longer EIS durations, particularly among trainees, who relied more heavily on visual input for comprehension. This reliance underscores the role of visual cues in supporting auditory-visual integration under lower cognitive demands [42]. This result also aligns with Zou et al. [13], who found that Eye-dominant interpreters produced the most accurate interpreting output. As speech rates increased, both groups exhibited a shift toward auditory-dominant processing, with declining EIS values reflecting efforts to synchronize listening and reading processes in real time. Professionals’ ability to maintain more balanced EIS durations supports Timarová et al.’s [45] observation that experienced interpreters excel in integrating multimodal inputs under complex conditions. In contrast, trainees struggled to achieve this balance, with reliance on visual input at slower speech rates resulting in greater variability and delayed delivery under faster rates. This highlights the importance of developing efficient cross-modal attention allocation strategies in interpreter training, helping students learn to dynamically adjust attention focus based on task demands.

Performance differences between professionals and trainees highlight the critical role of experience in optimizing resource allocation and temporal coordination. Professional interpreters consistently maintained better performance across all tasks, with holistic assessment scores remaining within an acceptable range (7.61 out of 10). They consistently rendered more sentences successfully, produced shorter sentence lengths, and demonstrated improved delivery fluency under all conditions. Consistent with Setton and Dawrant’s [15] observation, experienced interpreters excel in leveraging multimodal strategies to optimize performance, while trainees often struggle with cognitive overload in high-pressure scenarios [25]. The results also resonate with previous studies [5,12] on the importance of temporal synchronization in SI, as professionals’ shorter and more stable EVS durations reflect their ability to prioritize auditory input and dynamically adjust their pace. These insights highlight the importance of developing multimodal coordination strategies during interpreter training, particularly through simulating high-pressure scenarios, to prepare trainees for the cognitive challenges of real-world SI with text.

## Summary and conclusion

This study explores how variations in speech rate modulate multimodal processing traits of professionals and trainees, as reflected in their ear-eye-voice coordination patterns in SI with text tasks. Our analysis revealed a significant association between increased speech rate and a decrease in EIVS for both groups. Faster speech rates facilitated more synchronized auditory and visual processing, as evidenced by shorter temporal distances between listening, reading, and interpreting. While trainees exhibited a stronger reliance on visual input, particularly under slower speech rates, this reliance often disrupted their performance at higher speech rates. Such patterns highlight the need for targeted training to foster a balance between auditory and visual processing. In contrast, professionals displayed more consistent and efficient EIVS patterns, effectively integrating multimodal inputs to optimize performance. Despite declines in interpreting performance for both groups under increasing speech rates, professionals consistently outperformed trainees.

The findings illuminate cognitive mechanisms underlying temporal coordination patterns. Professionals’ ability to synchronize auditory and visual inputs demonstrates experience-driven adaptability, while trainees’ struggles reflect developmental challenges in achieving efficient multimodal coordination. Contrary to previously held associations between longer EVS and increased processing effort, our data suggest that professional interpreters strategically manage their EIVS to maintain output quality under challenging conditions. Such adaptability underscores experience as crucial for navigating multimodal interpreting complexities. Furthermore, this study highlights the value of EIVS as a dynamic measure of cognitive coordination in SI with text, offering insights into processing traits and coordination patterns linked to interpreting expertise and providing a framework for assessing professional development.

The findings offer practical implications for interpreter training. Training programs should implement graduated exposure to varying speech rates with visual support, enabling students to develop adaptive ear-eye-voice coordination skills. Importantly, visual support should reflect evolving interpreting technologies, from traditional materials to real-time ASR and MT outputs across various platforms used in offline, online or hybrid interpreting settings. Additionally, exercises should target strategic modality switching, helping students to flexibly shift between visual and auditory dominance based on task demands. Finally, trainees should be taught to strategically prioritize essential information while accepting planned omissions of secondary content to optimize processing resources. These approaches can help develop the cognitive flexibility, attentional control and resource management strategies that underpin expert performance in high-pressure scenarios [5,12,26,32]. Specific training activities might include eye-tracking supported exercises to optimize gaze patterns, scripted shadowing tasks to develop efficient ear-eye coordination, comparative analysis of professional versus student performances to highlight strategic adaptations, and progressive speed challenges simulating varying task demands. Through these targeted practices, interpreting training programs can better prepare students for the multimodal challenges of modern conference interpreting, where fast speech rates and visual materials are increasingly common.

Several limitations warrant consideration. Our study faced sample size imbalance between professionals and trainees—a common challenge in interpreting research due to limited professional participant availability [63]. Despite this constraint, the comparison offers meaningful insights into how experience shapes cognitive processing in SI. As interpreting practice increasingly incorporates diverse multimodal inputs, future research should expand to include scenarios with technology assistance, live subtitling, and ASR outputs to further explore connections between multimodal processing and performance. Investigating contextual factors such as text complexity, linguistic features, and cultural differences would provide a more comprehensive understanding of how interpreters manage multimodal demands. Moreover, advanced experimental designs, incorporating neuroimaging or longitudinal approaches, could offer deeper insights into the cognitive mechanisms underlying EIVS and inform more effective training methodologies.
